# 

**Published:** 1889-05-04

**Authors:** 


					No. 136, Vol. VL-
-May 4th, 1889.
[Registered at the General Post Office as a Newspaper.]
<j?> JMCy^. ij Ditto per Annum, 7s. 6d., post free.

				

